# On the Use of Opium in Continued Fevers

**Published:** 1850-01

**Authors:** 


*On the use of Opium in Continued Fevers.* By A. G. Henry, M. D.—(Boston Med. and Surg. Journal.)

For the last twelve years, opium, in *four* and *five* grain doses, has been my main remedy, in all forms of typhoid fever. In fact, when I use it at all in fever, it is in four or five grain doses. I claim to have demonstrated beyond all reasonable doubt, by a long and careful observation and experience, that while the maximum doses of the schools are of doubtful utility, and often prove injurious in fever, by increasing the dryness of skin, aggravating the pain in the head, &c., *a five grain dose* will, nineteen times in twenty, produce free perspiration, and relieve every unpleasant symptom. The notion that so generally prevails among the profession, that opium cannot be used to advantage in fever while there is determination to the brain, is certainly erroneous, if it is given in the doses which I recommend, unless there is *actual* inflammation of the membranes—and cases of this kind are extremely rare, in my opinion, Dr. Clutterbuck to the contrary, notwithstanding.

There is, I believe, a high degree of *irritation* in the brain, in our billious remittents, as a very general thing; and this irritation may ultimately terminate in actual lesion; but until this takes place, opium, in sedative doses, is, in my opinion, the appropriate remedy. I would not, however, theorize upon the subject. The practice which I advocate has been based upon facts, and I leave it to abler heads than mine to frame a philosophical theory to suit them. All I ask of my medical brethren is, to so far lay aside their preconceived opinions as to give the dose which I recommend a fair trial, when they resort to opium in fever as a remedy; and, my word for it, they will find the remedy, in four grains, not only safe, but far more beneficial than when given in one or two grain doses, at one or two hours intervals.

For example—I am called to a case of remittent fever in the morning. I find my patient with hot, dry skin, violent pain in the head and back, &c. If of a full plethoric habit, I would bleed, (but I very rarely resort to the lancet latterly); evacuate the stomach and bowels freely, and at bed-time I would give him five grains of opium, with ten or twelve of calomel, and direct him to drink *hot* tea for an hour or two. I should visit him in the morning with the confident expectation of hearing that he had rested well during the night, perspiring freely, and the pain in the head and back entirely relieved. I would then direct a purgative of salts and senna, or salts and cream of tartar, to be taken during the day. At night, I would give the calomel and opium again, and in the morning I should expect to find symptoms of slight ptyalism, with a full intermission, when *three* five-grain doses of quinine, with laxatives, would end the treatment. * *

The great power of opium as a remedial agent, when given in full *sedative doses,* is most striking and manifest in *dysenteric fever,* which so often prevails epidemically in your section of the Union. Nine times in ten, a single five-grain dose, combined with ten or fifteen of calomel, after blood-letting, will cure the disease, if resorted to within twenty-four or thirty-six hours after the attack.

